# MycoVarP: Mycobacterium Variant and Drug Resistance Prediction Pipeline for Whole-Genome Sequence Data Analysis

**DOI:** 10.3389/fbinf.2021.805338

**Published:** 2022-06-03

**Authors:** Sandeep Swargam, Indu Kumari, Amit Kumar, Dibyabhaba Pradhan, Anwar Alam, Harpreet Singh, Anuja Jain, Kangjam Rekha Devi, Vishal Trivedi, Jogesh Sarma, Mahmud Hanif, Kanwar Narain, Nasreen Zafar Ehtesham, Seyed Ehtesham Hasnain, Shandar Ahmad

**Affiliations:** ^1^ Department of Biochemical Engineering and Biotechnology, Indian Institute of Technology, Hauz Khas, New Delhi, India; ^2^ Department of Molecular Medicine, School of Interdisciplinary Sciences, Jamia Hamdard, New Delhi, India; ^3^ Inflammation Biology and Cell Signalling Lab, Safdarjung Hospital Campus, ICMR National Institute of Pathology, New Delhi, India; ^4^ ICMR Computational Genomics Centre, Informatics Systems and Research Management (ISRM) Division, Indian Council of Medical Research (ICMR), New Delhi, India; ^5^ School of Computational and Integrative Sciences, Jawaharlal Nehru University, New Delhi, India; ^6^ ICMR-Regional Medical Research Centre, Dibrugarh, India; ^7^ Department of Biosciences and Bioengineering, Indian Institute of Technology-Guwahati, Guwahati, India; ^8^ Department of Pulmonary Medicine, Guwahati, India; ^9^ New Delhi Tuberculosis Centre, New Delhi, India; ^10^ Department of Life Sciences, Sharda University, Greater NOIDA, India

**Keywords:** drug resistance, drug susceptible, lineage prediction, MDR-TB, *Mycobacterium tuberculosis*, PE-PPE/PGRS family, single-nucleotide variants

## Abstract

Whole-genome sequencing (WGS) provides a comprehensive tool to analyze the bacterial genomes for genotype–phenotype correlations, diversity of single-nucleotide variant (SNV), and their evolution and transmission. Several online pipelines and standalone tools are available for WGS analysis of *Mycobacterium tuberculosis* (*Mtb*) complex (MTBC). While they facilitate the processing of WGS data with minimal user expertise, they are either too general, providing little insights into bacterium-specific issues such as gene variations, INDEL/synonymous/PE-PPE (IDP family), and drug resistance from sample data, or are limited to specific objectives, such as drug resistance. It is understood that drug resistance and lineage-specific issues require an elaborate prioritization of identified variants to choose the best target for subsequent therapeutic intervention. Mycobacterium variant pipeline (MycoVarP) addresses these specific issues with a flexible battery of user-defined and default filters. It provides an end-to-end solution for WGS analysis of *Mtb* variants from the raw reads and performs two quality checks, *viz*, before trimming and after alignments of reads to the reference genome. MycoVarP maps the annotated variants to the drug-susceptible (DS) database and removes the false-positive variants, provides lineage identification, and predicts potential drug resistance. We have re-analyzed the WGS data reported by Advani et al. (2019) using MycoVarP and identified some additional variants not reported so far. We conclude that MycoVarP will help in identifying nonsynonymous, true-positive, drug resistance–associated variants more effectively and comprehensively, including those within the IDP of the PE-PPE/PGRS family, than possible from the currently available pipelines.

## 1 Introduction

The emergence of resistance in *Mycobacterium tuberculosis* (*Mtb*) strains against drugs contributes significantly to high mortality in tuberculosis patients. According to the global tuberculosis (TB) report-2020, drug-resistant TB became a major public threat, and half a million people developed *rifampicin* resistance, out of which 78% developed multidrug resistance to TB (MDR-TB) around the globe (WHO report, 2020; Koegelenberg et al., 2021). The major TB burden countries are India, with 27%, followed by China (14%) and the Russian Federation (8%). In 2019, 3.3% of new TB cases were recorded with previously treated MDR/RR-TB (17.7% cases) (WHO report, 2020; Koegelenberg et al., 2021; World Health Organization, 2021). Recent studies have shown that *Mtb* acquired resistance against bedaquiline and delamanid, the two new drugs recently approved for MDR/extreme drug resistance (XDR) to TB (Bloemberg et al., 2015; Degiacomi et al., 2020; Yao et al., 2021). Thus, the main challenge for the treatment of TB is the ever-evolving mechanism of *Mtb* to evade existing and recently approved drugs. Whole-genome sequencing (WGS) identifies sequence information of variants responsible for drug susceptibility (DS) and the emergence of drug resistance (DR) (Dohál et al., 2020; Lam et al., 2021). WGS helps to understand the evolution, transmission dynamics, outbreak investigation, and lineage classification (strain typing) to infer resistance and the distribution of the bacterial genomes of *Mtb* (Advani et al., 2019; Gygli et al., 2019). WGS studies have surged after 2010 to achieve the goal of the end TB strategy (Cohen et al., 2019) (Supplementary Figure S1). Computational analysis of variants and their sequence-level differences are typically performed by applying a series of well-established computational tools for different steps involved in variant calling. Researchers have widely optimized the sequence and selection of computational tools to develop analysis pipelines for specialized tasks with best outcomes. However, these pipelines cannot be used by less technically oriented scientific communities as the user wants to change some parameters in the pipeline, but MycoVarP is more focused on variant prioritization with several output options. The genomics analysis user can change the script as per the requirement in the bash script.

There are several preexisting pipelines and web servers to identify/predict single-nucleotide variants (SNVs) in *Mtb*, for example, WGS-TB-RESISTANCE (Cingolani et al., 2012). However, most of the WGS analysis pipelines are based on a rigid and standardized protocol for SNV identification and drug resistance prediction (Meehan et al., 2019. Currently, the WGS data can be analyzed by TB Profiler (Phelan et al., 2019), PhyResSE (Feuerriegel et al., 2015), MUBII-TB-DB (Flandrois et al., 2014), MTBseq (Kohl et al., 2018), CASTB (Comprehensive Analysis Server for the *Mycobacterium tuberculosis* complex) (Iwai et al., 2015), unified analysis variant pipeline (UVP) Relational Sequencing Tuberculosis Data Platform (ReSeqTB), and other standalone tools such as Mykrobe predictor (Bradley et al., 2015; Hunt et al., 2019) and KvarQ (Steiner et al., 2014; Dohál et al., 2020), etc (Supplementary Tables S1, S2). As Meehen et al., 2019 stated that not only more standardization is required but also pipelines should have a flexible and dynamic framework in which the rapidly changing status of drugs can be effectively incorporated. As new MTB cases with new lineages and novel mutations are being constantly reported and became an issue of more concern around the globe, DR to MDR/XDR cases are also growing rapidly, leaving the static and highly “standardized” protocols inadequate. In view of this, the MTB pipelines need to be standardized by keeping them sufficiently flexible for updates at the level of databases and analytics.

Next-generation sequencing (NGS) of *Mtb* has been utilized to reveal their relationship between its genome, transcriptome, methylome, identification of subspecies, associated lineages, transmission inference, and possible transmission within the host and interspecies. The molecular clock of *Mtb* has been delineated using WGS, which gives insights for deciphering the accurate phylogenetic relationship among different mycobacterial strains (Menardo et al., 2019). This approach has proved invaluable to understand the evolution of extensively drug-resistant (XDR) tuberculosis variants over a long period and understand the genes and intergenic regions which lead to DR (Rancoita et al., 2018; Brown et al., 2019; Gomez-Gonzalez et al., 2019). Several studies have involved machine learning (ML) and statistical models to predict DR and uncover putative phenotype-associated mutations (Chen et al., 2019; Deelder et al., 2019). WGS has explained the transmission dynamics of *Mtb* within the patient and its transfer to secondary cases (Séraphin et al., 2019). The epidemiological, evolutionary, and relapse/reinfection of *Mtb* has been unraveled using WGS (Folkvardsen et al., 2017; Brown et al., 2019). ML-based methods of WGS data analysis remain the backbone of modern variant identification and DR. ML methods such as Shanmugam’s classification tree and gradient-boosted tree have been applied to the WGS data to predict existing and novel DR variants (Chen et al., 2019; Deelder et al., 2019). In summary, WGS studies, particularly with the help of various ML models, have helped to understand the DR, XDR, and MDR; genetic heterogeneity; and region-specific variations including those in the Indian population (Advani et al., 2019; Shanmugam et al., 2019). One caveat observed from the analysis of these studies is that they often produce inconsistent outcomes. For example, studies focusing on the comparative analysis of WGS data sets and drug susceptibility have shown that there is variability in the detection of the level of resistance. For example, resistance predictions based on different databases such as PhyResSE and TB-Profiler (Faksri et al., 2019) have produced different and, sometimes, contradictory annotations of novel XDR variations. Substantial progress has been made in *Mycobacterium* spp., genome analysis, and the issues addressed in the available literature range from the GC-enrichment, repetitive genes, mobile genetic elements, and intrinsically disordered region containing proteins such as PE/PPE-PGRS and ESAT-6. PE/PPE-PGRS constitute 10% of the *Mtb* genome and have repetitive regions that belong to the intrinsically disordered region (IDP) protein family (Naz et al., 2019). Many of these properties remain difficult to analyze using the readily available software or by the use of web-based public resources (Meehan et al., 2019). Realizing this, there has been an effort to develop a worldwide consortium of resistance databases such as ReSeqTB and Comprehensive Resistance Prediction for Tuberculosis: an International Consortium (CRyPTIC) project (CRyPTIC Consortium et al., 2018; Rancoita et al., 2018). These consortia and platforms have focused on specific issues of drug resistance and variants, for example, CRyPTIC deals with the minimum inhibitory concentrations for the drugs and genetic variants. From the practical point of view, there is no consensus on a method to measure a pathogen’s genomic variation, and studies frequently use different sequence quality control measures, mapping algorithms, and variant calling as per the ploidy of the organism and apply variant filters to remove false positives and other irrelevant variants. Such parameterization is critical to producing reliable targets for drug discovery in the case of resistant variations in *Mtb*. Considering these shortcomes, we have incorporated the state-of-the-art knowledge about *Mtb*, best practices of variant calling, and DR quantification into a comprehensive pipeline for WGS data analysis, which can address the limitations of all the available computational tools and draw upon their best use-case scenario. The primary advance introduced in this study, therefore, is by way of integrating multiple filters that help to prioritize and select SNVs based on minor allele frequency (MAF), removal of the IDP family of PE/PPE genes, and removing the false-positive variants. PE-PPE (IDP family) and repetitive regions have a high rate of mutation due to disordered regions. Therefore, it is difficult to sequence using short-read sequencing. The technical and use-case discussion of this tool MycoVarP, Mycobacterium Variant and Drug Resistance Prediction, are provided in the following sections. As stated, these methods rely on pipeline-specific constant parameters for analysis and do not provide the flexibility to tune them according to user requirements in view of the quality of the raw sequencing data and diversity in the strains of Mycobacterial isolates from different geographical regions. In the following sections, we review these issues specifically and present a novel pipeline to analyze lineage and DR patterns of clinical strains from WGS data. In the proposed pipeline, called MycoVarP, a number of steps have been automated based on best clinical outcomes, for example, SNVs related to repetitive regions have been excluded and rigorous downstream filtering after variant calling is included (more details in Supplementary Table S1). This tool is developed for short-read sequencing of WGS of *Mtb*. These automation steps, together with clinically supported outcome analysis, are likely to help in the lineage-specific drug discovery of *Mtb* strains.

## 2 Materials and Methods

### 2.1 Description of MycoVarP for WGS Analysis

**FIGURE 1:**
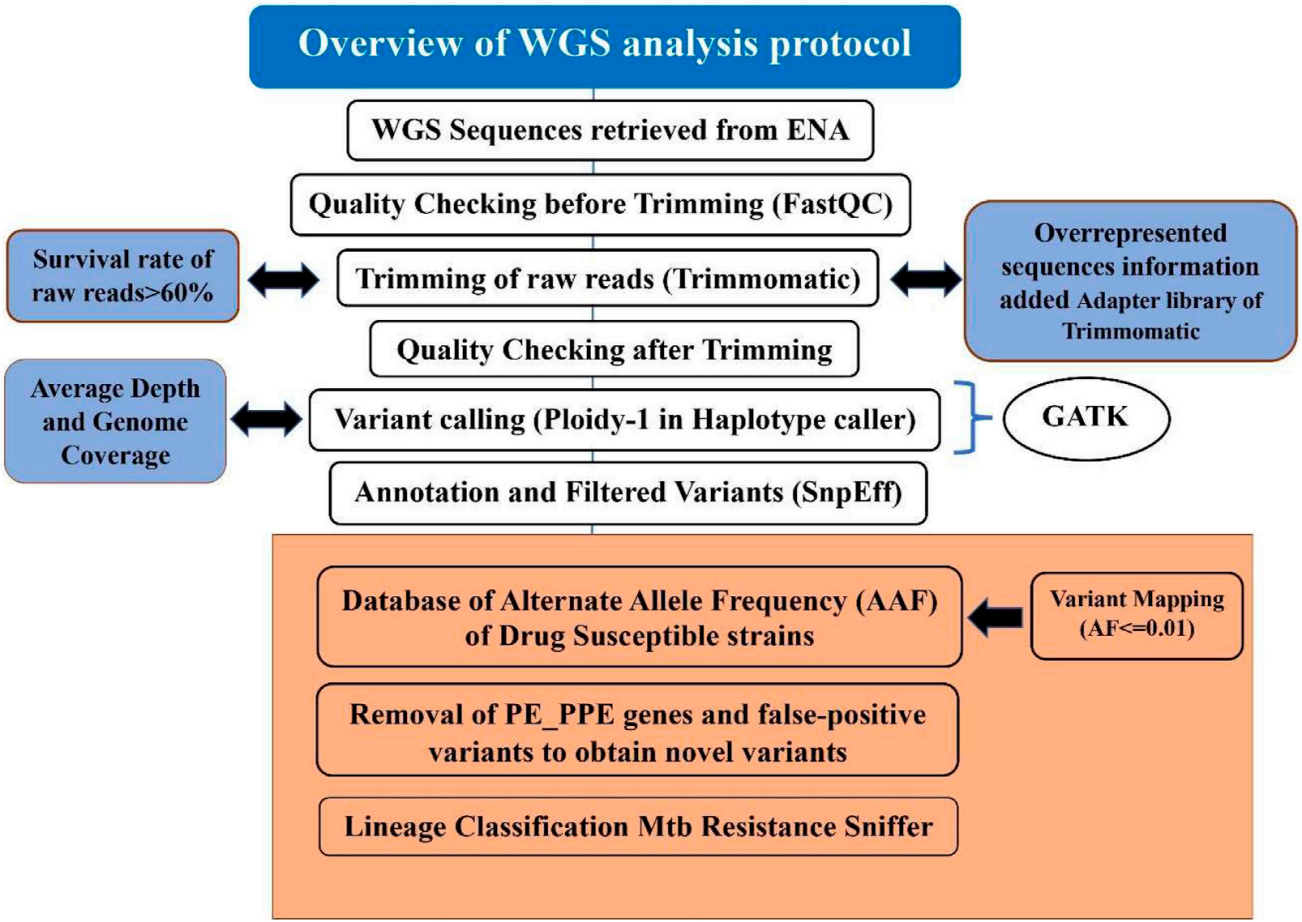
Step-by-step procedure of the MycoVarP pipeline for *Mtb* whole-genome approach. The text represented in the workflow shows the steps which are not included in any other existing WGS *Mtb* pipelines. The box present in orange shows the steps involved in variant prioritization such as mapping the query sample against the drug-susceptible database and removal of intrinsically disordered region (IDP) protein family-like PE/PPE genes and false-positive variants.

The detailed steps of WGS analysis as used in MycoVarP have been given in Figure 1, and its critical comparison with alternatives has been discussed in detail in the following sections.

#### 2.1.1 Quality Check (QC) and Trimming of Raw Reads

It is important to filter out the low-quality data files that would impact the downstream results by introducing noise or systematic bias to the analyzed dataset. In MycoVarP, we provide a quality check at two stages, *viz*., 1) for the selection of raw reads by quality check (QC) and 2) at the sequence alignment level. Quality is checked for G + C content, INDELs/repetitive regions, duplicates adapters, overrepresented sequences, genome coverage, and depth of coverage (Dohál et al., 2020).

The most popular tool for quality control is Fastqc (Andrews, 2012). It is faster than other QC tools, such as PRINSEQ, FASTXToolkit, and NGS QC Toolkit. Although a new version of QC tool Fastp is also available, a number of limitations have been reported (Liu et al., 2020). We have, therefore, selected FastQC as a primary tool for quality check in our pipelines. FastQC analysis includes base quality, GC content, sequence length distribution, duplication levels, overrepresented sequences, adapters, and K-mer content. Before submitting the data to this pipeline, a user has to check the quality of samples manually by using FastQC for better results (MycoVarP_Documentation file). The quality checks of some of the parameters such as adapter contamination, overrepresented sequences, and per-base sequence contents can be improved by trimming. In our pipeline, the raw reads are trimmed using Trimmomatic (Bolger et al., 2014). The parameters in Trimmomatic can be adjusted by the user according to the quality of the data to obtain better results. The minimum read length for all samples is kept at 25 bases. The overrepresented sequences present in the raw reads in addition to adapters are added to the adapter library of Trimmomatic so that they can be removed from reads while trimming. A total of 6,750 adapter sequences are added to the Trimmomatic adapter library which are defined as TruSeq Adapter (Index 1, 2, 3, 4, 5, 6, 7, 8, 9, 10, 11,12,13, 14, 15, 16, 17, 18, 19, 20, 21, 22, 23, 25, and 27), Illumina PCR Primer (Index 9 and 10), and no-hit adapters. Since there is noise in the samples, CROP and HEADCROP can be used for trimming (Available in Supplementary Material as MycoVarP_Documentation file). The reads with a low survival rate after trimming will yield poor results. Therefore, it is better to discard samples with low survival rates (Del Fabbro et al., 2013). In this pipeline, raw reads which showed a survival rate of less than 60% are automatically excluded. The remaining trimmed reads can be rechecked for their quality and then considered for variant calling.

#### 2.1.2 Variant Calling

Variant calling is the identification of SNV from the WGS data, which confers a disease predisposition and potentially DR. SNVs obtained from WGS may be the same as reported in earlier studies, or novel variants may be obtained depending on the type of the study and sample. Variant calling involves alignment of each of the trimmed reads to the reference genome and marking duplicates and variant identification is depicted in the flow chart (Figure 1). In the present pipeline analysis, the trimmed reads are mapped with the *Mtb* H_37_Rv reference genome (GenBank GCA_000195955.2) using BWA (Li et al., 2009) to obtain an alignment file. Genome coverage and average read depth were calculated with SAMTools which are important parameters for the quality of the reads. When sample reads mapped with the reference genome, it covered the genome <90%; then, the sample will be excluded from the study. If the average depth for the sample is < 100, then the samples with low genome coverage and average depth were automatically discarded. After the alignment, GATK (Van der Auwera and O'Connor, 2020) suite was used to identify variants (De Summa et al., 2017) by defining the haplotype caller module of GATK in the haploid mode as *Mtb* is a prokaryote.

To know the functional importance of genomic variants, it is used for annotating and prioritizing. It is the most important component of any NGS pipeline that needs to be developed. Variants need to be annotated as per their genomic position for functional information, population frequency, and allele affect. We then included standard methods and most reliable tools, such as SnpEff (Cingolani et al., 2012) and ANNOVAR (Wang et al., 2010), to annotate the variants in the pipeline.

#### 2.1.3 Variant Prioritization

The criteria for SNV prioritization from raw data are based on their effect on the encoded protein and their ability to narrow down to only the most relevant variants (SefidDashti and Gamieldien, 2017). The allele frequency of certain SNVs among the samples may play a role in the development of DS and DR. Therefore, variants are filtered based on their allele frequency against the in-house prepared WGS-based allele frequency DS database which is constructed using the samples reported to be susceptible and contains the allele information from ReSeqTB, PhyResSE database, and review of the literature (Feuerriegel et al., 2015; Ezewudo et al., 2020) (Provided in the Pipeline supporting files: AFF_MTB.vcf) for mapping. Minor allele frequency is calculated for the DS variant file (drug susceptible file contains 2,832 samples that constitute 118,869 variants) and variants having frequency ≤0.01 are filtered. This filtration provides the variants which are present in the population in low frequency and maybe the precursors that lead from DS to DR strains. This will scrutinize the number of variants and the variants in which higher frequency in the DS strains has been removed. Furthermore, silent mutations are screened from the variant files as these mutations do not lead to any change in the bacteria, but the user can be kept as per the requirement to understand the impact of these mutations during the analysis. The mapping of SNVs obtained after variant calling may have false-positive variants which need to be excluded from the data. The FPfilter tool has better accuracy to remove false-positive variants which is better than its alternative, namely, GATK-Hard Filters (Tan et al., 2020). This pipeline also takes care of repetitive and IDP regions of PE/PPE/PGRS proteins. These proteins cover up to 10% of the *Mtb* proteome and may interfere in analysis. Therefore, we have made prioritizing in the pipeline to identify these regions and also remove variants found in these regions. The variants which code for the repetitive and intrinsically disordered regions of PE/PPE proteins are segregated as these may interfere with the analysis (Zeng et al., 2018). The filtered variant file is further checked for the minimum sequencing depth (DP) supporting a genotype with the threshold value for DP as 10 (Lee and Pai, 2017). After obtaining the most relevant variants, the samples are analyzed using ANNOVAR to obtain the variants which are present in high frequency and distributed among most of the samples (Wang et al., 2010). The variant distribution across the dataset is graphically presented by Maftools (Mayakonda et al., 2018) utility of the R package.

#### 2.1.4 DR and Lineage Prediction

In our pipeline, the Resistance Sniffer program (Muzondiwa et al., 2020) was used for DR and lineage prediction. It allows the analysis of sequence datasets in multiple file formats such as gb, .gbk, .gbf, .gbff, .fasta, .fas, .fna, .fst, .fa, .fnn, .faa, and .vcf files obtained at different stages of genome sequence completion (Muzondiwa et al., 2020). It has included information on DR trials concerning the following antibiotics: amikacin (AMK), capreomycin (CM), cycloserin (CS), ethambutol (EMB), ethionamide (ETH), isoniazid (INH), fluoroquinolones (FLQ), kanamycin (KAN), ofloxacin (OFL), para-amino salicylic acid (PAS), pyrazinamide (PZA), rifampicin (RIF), and streptomycin (SM). The SNPs were processed using the diagnosis key, which consists of a catalog of clade-specific polymorphisms and genetic determinants of antibiotic resistance and genes. A resistance R-value greater than 0.75 predicts the strain to be resistant against the given antibiotic with a likelihood of 55% or higher. If the R-value is less than 0.3, the strain is deemed sensitive to the antibiotic with a likelihood of 55% or higher. The entire flowchart of the pipeline is illustrated in Figure 1.

## 3 Results and Discussion

### 3.1 Case Study

In order to demonstrate and assess the proposed pipeline on a real data set, we carried out the comparative analysis of WGS data from the EBI project ID PRJNA379070 (https://www.ebi.ac.uk/ena/browser/home). This project contains clinical samples of *M. tb* from India that showed genetic heterogeneity and variation specificity based on the geographical region (Advani et al., 2019). The dataset has been analyzed using the MycoVarP pipeline and for comparison with a similar tool Mykrobe to know variants and resistance of the samples against the antibiotics. The following steps were involved in this analysis.

### 3.2 QC and Trimming of Raw Reads

To generate a quality report, 200 samples were submitted to the FastQC tool and six samples were discarded due to errors such as per-base sequence quality (lower quartile for base <5 or median for base <20), per-sequence quality score (mean quality is <20 with 1% error rate), per-base sequence content (difference of A and T or G and C is >20% in any position), per-base GC content: (GC content of base >10% with mean of total GC), per-sequence GC content: (normal distribution >30% of the reads), per-base N Content: (N content >20% of the reads), sequence length distribution: (if any have zero length), duplicate sequences (if non-unique sequences make up >50% of the total sequence), overrepresented sequences (>1% of the total sequence reads), and overrepresented K-mers(enrichment of k-mer >10 fold at any individual base position). Trimming of the 194 samples was carried out using Trimmomatic-0.39, and 36 samples were discarded due to low survival score <60. For each read, the survival rate was checked so that the samples with good quality were considered for further variant calling analysis.

### 3.3 Variant Calling and Filter Analysis

The 164 samples are submitted to the MycoVarP pipeline using an in-house developed shell script which is given in an additional file.

### 3.4 Results of MycoVarP and Comparison With Existing Data and Pipeline

In variant calling analysis, 136 samples passed all the filters, whereas 28 samples are discarded due to low average depth <10, genome coverage <90%, and other stringent filters applied in MycoVarP. Advani et al. (2019) reported 18,970 nonsynonymous SNPs and 3,052 insertions and 2,739 deletions were identified in 161 samples when compared with the H_37_Rv genome (Table 1).

**TABLE 1:** Number of variants observed in the published data of Advani et al. (2019) and the MycoVarP pipeline.

		MycoVarP pipeline	
Type of variant	Advani et al. (2019)	Variants after removing synonymous variants	Variants after filtration
Nonsynonymous variants	18,970	18,435	931
INDELs	5,791	7,484	1,549

After mapping the annotated vcf file with the drug-susceptible database, we obtained 25,919 variants, out of which 18,435 are missense variants and 7,484 are INDELs (Supplementary Tables S3, S4). After the removal of the low DP (for each variant), false-positive filter, alternative allele frequency and removal of PE/PPE of the IDP protein family, the total number of variants remaining in the samples was 2,480, out of which the missense variants were 931 and 1,549 variants belonging to INDEL SNPs (Supplementary Tables S5, S6).

Advani et al. (2019) had evaluated the prevalence of fluoroquinolone resistance among isolates sequenced in their study by looking at gyrA and gyrB gene mutation frequencies. They identified 26 SNVs in the gyrA gene (45% of the samples), out of which 10 are known to cause resistance to fluoroquinolones. In addition to this, S95T in gyrA was also reported. However, when we carried out the analysis by MycoVarP, we found 15 sites for the SNVs of gyrA. These variants were present in all the 136 samples (100% samples). Furthermore, the variants for KatG and rpoB genes were also observed in the analysis carried out by Advani et al., that is, Mykrobe and MycoVarP. Both the mutations were present in all the samples in our analysis. In addition, we found one more mutation in rpoB protein at the 170th position which is V170F (Table 2). In this variation, Val is replaced by Phe which is having a long side chain, and this may interfere with the overall structural stability of the protein (Capriotti et al., 2005); we have observed this change in one of the clinical samples of Advani et al., 2019, and this mutation was not reported by them.

**TABLE 2:** SNVs with a high mutation rate in katG and rpoB were reported by Advani et al., 2019. The same mutations were observed and correlated using Mykrobe and MycoVarP pipelines. The results obtained by MycoVarP are supported by the existing pipeline and published data.

Resistance gene	Advani et al., 2019 (BWA, GATK, pINDEL, and in-house .py script)	Mykrobe pipeline	MycoVarP pipeline
katG	S315T (45% samples)	S315T	S315T (100% samples)
rpoB	S450L (28% samples)	S450L	S450L, V170F (100% samples)

The variants which are reported to be resistant against BDQ were also found in the analysis (Battaglia et al., 2020). In addition, we have analyzed the variants which were more frequently observed among most samples (Table 3). Most of the proteins in which these variants were observed are transporter proteins, involved in cell envelope synthesis, fatty acid biosynthesis, amino acid metabolism, methyltransferase, glycoside hydrolase enzyme, and uncharacterized proteins. Therefore, these are the possible driving forces that lead to the development of MDR in *Mtb*. By targeting these proteins, we may be able to design effective drugs against the MDR strains of *Mtb*.

**TABLE 3:** Additional list of proteins identified using MycoVarP compared to those identified by Advani et al. (2019).

Category of protein	Gene ID	Gene name	Protein name	Number of altered samples
Miscellaneous	Rv 2081c	Rv 2081c MTCY49.20c	Uncharacterized protein Rv 2081c	112
Miscellaneous	Rv0071	Rv0071	Possible maturase	92
Miscellaneous	Rv2264c	Rv2264c	Conserved hypothetical proline-rich protein	86
Miscellaneous	Rv0021c	Rv0021c	Uncharacterized protein	65
Cell signaling	Rv0592	mce2D Rv0592	Mce-family protein Mce2D	55
Methyltransferase	Rv3919c	rsmG gidB Rv3919c MTV028.10c	Ribosomal RNA small subunit methyltransferase G	50
Fatty acid biosynthesis	Rv1527c	pks5 Rv1527c LH57_08,370	Mycocerosic acid synthase–like polyketide synthase (MAS-like PKS)	37
Fatty acid biosynthesis	Rv1661	pks7 Rv1661	Probable polyketide synthase Pks7	35
Fatty acid biosynthesis	Rv3800c	pks13 Rv3800c	Polyketide synthase Pks13	33
Cell wall biosynthesis	Rv3795	embB Rv3795 MTCY13D12.29	Probable arabinosyltransferase B	31
Miscellaneous	Rv0395	Rv0395	Uncharacterized protein	30
Fatty acid biosynthesis	Rv1662	pks8 Rv1662	Probable polyketide synthase Pks8	30
Miscellaneous	Rv1233c	Rv1233c	Conserved hypothetical membrane protein	29
Amino acid metabolism	Rv2531c	Rv2531c	Probable amino acid decarboxylase	29
Glycoside hydrolase enzyme	Rv3401	Rv3401 MTCY78.27c	Uncharacterized glycosyl hydrolase Rv3401	29
Intermediary metabolism and respiration	Rv2918c	glnD Rv2918c MTCY338.07c	Bifunctional uridylyltransferase/uridylyl-removing enzyme (UTase/UR) (bifunctional [protein-PII] modification enzyme) (bifunctional nitrogen sensor protein) [includes: [protein-PII] uridylyltransferase (PII uridylyltransferase) (UTase)	29
DNA synthesis	Rv0570	nrdZ Rv0570	Vitamin B12-dependent ribonucleoside-diphosphate reductase (B12-dependent RNR)	28
Secretory system	Rv3876	espI Rv3876	ESX-1 secretion-associated protein EspI	28

### 3.5 Drug Resistance Results From the Proposed Pipeline

**FIGURE 2:**
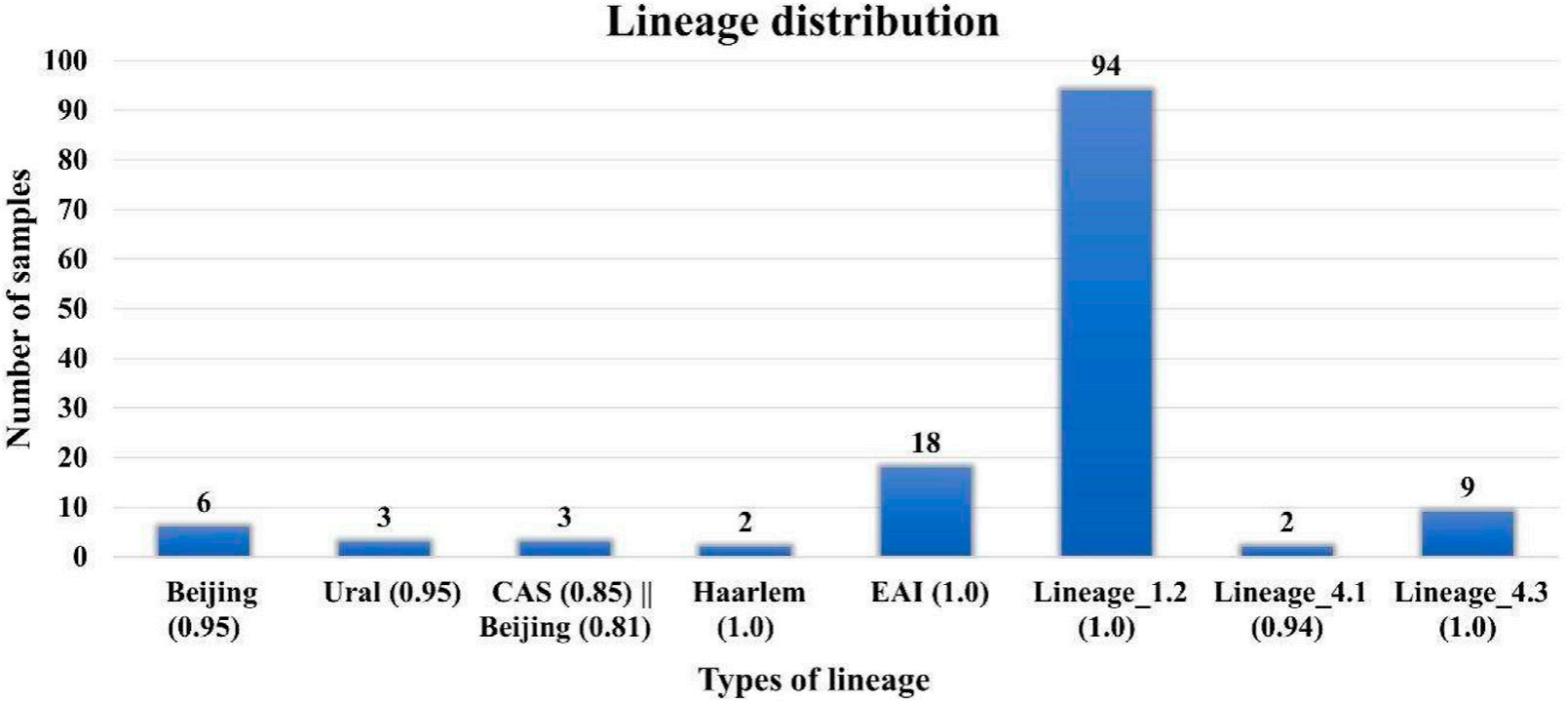
Lineage distribution of 137 samples where Lineage 1 (Indo-Oceanic) found in the Indian Ocean; Lineage 2: East Asia, including Beijing; Lineage 3 East Africa and India (Central Asian (CAS)/Delhi); Lineage 4 (Euro-American) Africa, Europe, and America, American–Mediterranean (LAM), Haarlem, X type, and T families.

DR analysis results were similar to those reported earlier by Advani et al. However, in our lineage analysis, we found that specific lineages, that is, Beijing (0.95), Lineage 1.2, EAI (1.0), CAS (0.86) || Beijing (0.82), Haarlem (1.0), CAS (0.85) || Beijing (0.81), Ural (0.95), and Lineage 4.1 and 4.3 are different from those in the previous reports (Figure 2) (Supplementary Table S7). Lineage analysis of 137 samples revealed mostly 69% of the samples belong to the Beijing strain (100 samples), and then 13% are Lineage 3 (18 samples) and 7% are Lineage 4 (10 samples) and other lineages (Figure 2). We conclude that the proposed pipeline has been able to identify variations and lineages previously missed or misunderstood by previous reports aiming to perform similar tasks.

### 3.6 Discussion and Challenges in Variant Analysis

WGS pipelines in the public domain have so far helped us to identify the variants, predict their lineage, and DR association of the variant. However, there is a need to apply variant filters and prioritization steps to narrow down the most relevant variants from the raw data . vcf file (De Summa et al., 2017). These filters may include a cutoff for the allele frequency in the samples; application of hard filters such as Base-QRankSum, ClippingRankSum, DP, MQ, GQ, MQRank-Sum, and ReadPosRankSum; removal of false-positive variants present in the vcf file; and removal of synonymous variants (De Summa et al., 2017).

#### 3.6.1 Selection of the Reference Genome for Alignment

There is a need to select the reference genome according to the geographical region of the isolate. In most of the WGS analysis pipelines, the *Mtb* strain H_37_Rv genome has been used as the reference genome. However, there are other complete *Mtb* genome sequences available that can be explored to analyze different strains having diverse lineages and accordingly, the most appropriate lineage reference genome can be defined.

The variants obtained from WGS analysis of human sequences have well-defined Rsid (reference SNP cluster ID) which is present in the single-nucleotide polymorphism database (dbSNP) and accession numbers for variations observed in human diseases available in the ClinVar database. However, in the case of *Mtb*, there is large WGS data available, still the SNPs of its WGS data do not have ID like the human genome. Therefore, there are no standardized databases of SNPs that can be used to recalibrate and annotate variants while variant calling. Variant recalibrator takes the overlap of the training/reference/truth resource sets and query call set. The VQSLOD (for variant quality score log-odds) score is added to the INFO field of each SNP and checks whether the obtained SNP is true or false under the trained Gaussian mixture model. Therefore, there is a need for a standardized *Mtb*_reference.vcf file for the recalibration step.

#### 3.6.2 Need of the Standardized File Containing Importance of Repetitive Regions

*The Mtb* H_37_Rv genome consists of 99 PE, 69 PPE, and 61 PE-PGRS (polymorphic GC-rich) genes which have a variety of functions (Akhter et al., 2012; Zumbo et al., 2013; Grover et al., 2018; Sharma et al., 2020) The homologous recombination between the genetic material evidenced and shaped the evolution of these genes (Gey van Pittius et al., 2006) with the long regions of ESX. There is a need for in-depth studies to understand the difference between genetic polymorphism within several clinical isolates compared to antigenic variance. McEvoy et al. (2012) stated that nonsynonymous SNP mutations occurred more in the PE/PPE genomic regions than other regions which lead to genome plasticity; this property of PE/PPE proteins can be attributed to the intrinsically disordered region present in these proteins. Studies explained the importance of the IDP family of PE/PPE protein in pathogenicity and should be considered potential drug targets, but a better understanding of these proteins is required in the genomic-level studies. There is a need to focus on understanding the duplication levels in the genomes with similar genes; repetitive gene families; next-generation sequencing data analysis with alignment algorithms that discard the genes automatically; mapping with the reference genome and clinical strain genome pattern; and restricting the reliable PE/PPE sequence data size.

To overcome this, we need well-defined and standardized sequencing platforms that yield long-length reads with advanced alignment based on mathematical algorithms. Detection of the single PE/PPE overexpression is very difficult using conventional methods because of functional redundancy.

#### 3.6.3 Standardization of the Tools According to the Genome Diversity

The NGS techniques revealed a heterogenous collection of phylogeographical data. How this genomic diversity contributes to research-based clinical findings is relatively unexplored. There are seven lineages reported for *Mtb*. Ngabonziza et al. (2020) reported the lineage L8 sister clade by genome-based phylogenetic reconstruction to the known MTBC lineages. This lineage has diverged by the loss of the *cobF* genome region that encodes Precorrin-6A synthase which is required for the biosynthesis of cobalamin/vitamin B12 (Ngabonziza et al., 2020). A clear understanding of TB infection and its evolution is possible by exploring the molecular clock of the genome by finding the genetic variants which are involved in the DR mechanism and divergence of strains/lineages (Veyrier et al., 2011; Reva et al., 2015). Previous studies revealed that DR is majorly linked with the Beijing strain with lineage-specific mechanisms (Toungoussova et al., 2002; Liu et al., 2020). Earlier studies reported a lot of diversity in the DR mechanism pattern when compared with non-Beijing isolates (Lari et al., 2006). Phylodynamic analyses need to be carried for a better understanding of the evolutionary trend of SNPs that unravel the mechanism involved in DR. There is a need to reconsider the reference genome and analyze the available complete genome available on the NCBI. Furthermore, there is a need to define parameters as per the strains and lineage of *Mtb.*

WGS data can be used to detect the phylogenetic distance among the diverse isolates which will give a clear understanding of the potential impacts of mutations. Whole-genome sequencing (WGS) analysis of *Mtb* is in its initial stages. In the Indian context, the population is diverse and TB burden is high. However, there are few studies on the North and South Indian groups. Therefore, there is a need to explore the clinical data of WGS from different parts of India. The TORCH consortium is one of the capacity-building initiatives for TB research in low-income countries such as Ethiopia. Such initiatives are required to be implemented in India.

## 4 Conclusion

In the present work, WGS analysis steps have been carried out with stringent quality parameters to obtain significant results. The steps involved in the WGS analysis have been explained in detail in the abovementioned text, and additional information has been added to the adapter library of Trimmomatic. Our results on a case study indicate that the proposed pipeline can detect many variants that are missed from current public reports, and thereby a better treatment strategy informed by accurate identification of DS can be developed. Being a user-friendly and mostly fully automated pipeline, we believe that MycoVarP can be used to carry out WGS studies on the clinical data of *Mtb by* researchers less familiar with computational nuances*.*

## Data Availability

The original contributions presented in the study are included in the article/Supplementary Material, further inquiries can be directed to the corresponding author.
