# Supplementary material for: MycoVarP: Mycobacterium Variant and Drug Resistance Prediction Pipeline for Whole-Genome Sequence Data Analysis
Source: Front Bioinform. 2022 Jun 3;1:805338. doi: 10.3389/fbinf.2021.805338 (PMC9580932; doi:10.3389/fbinf.2021.805338)
Supplement: Supplementary file 1 [file DataSheet1.zip › Supplementary Material MycoVarP_Documentation File.docx]

**MycoVarP Documentation File.** Input and Output documentation of MycoVarP

**Input page of MycoVarP**


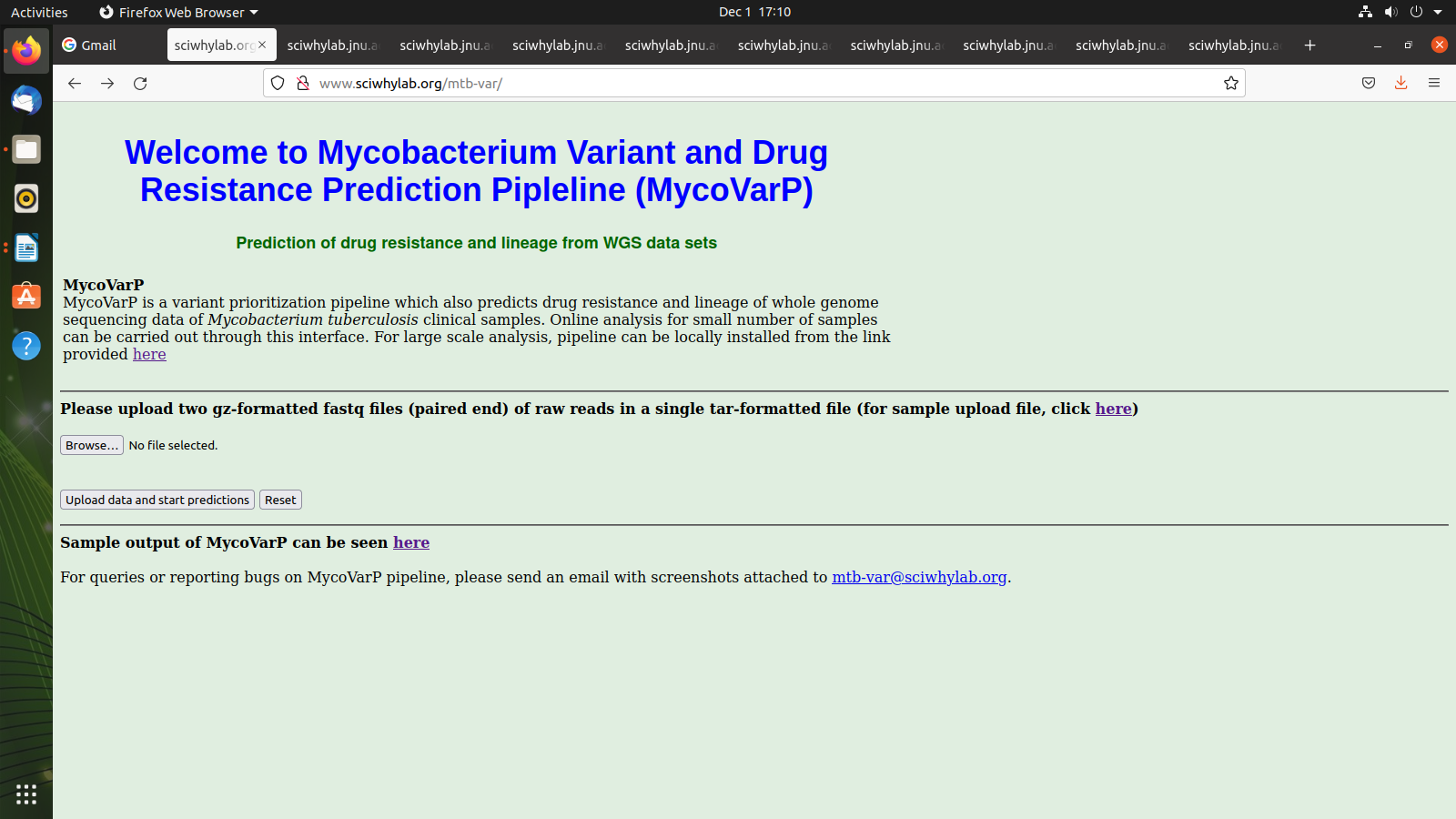


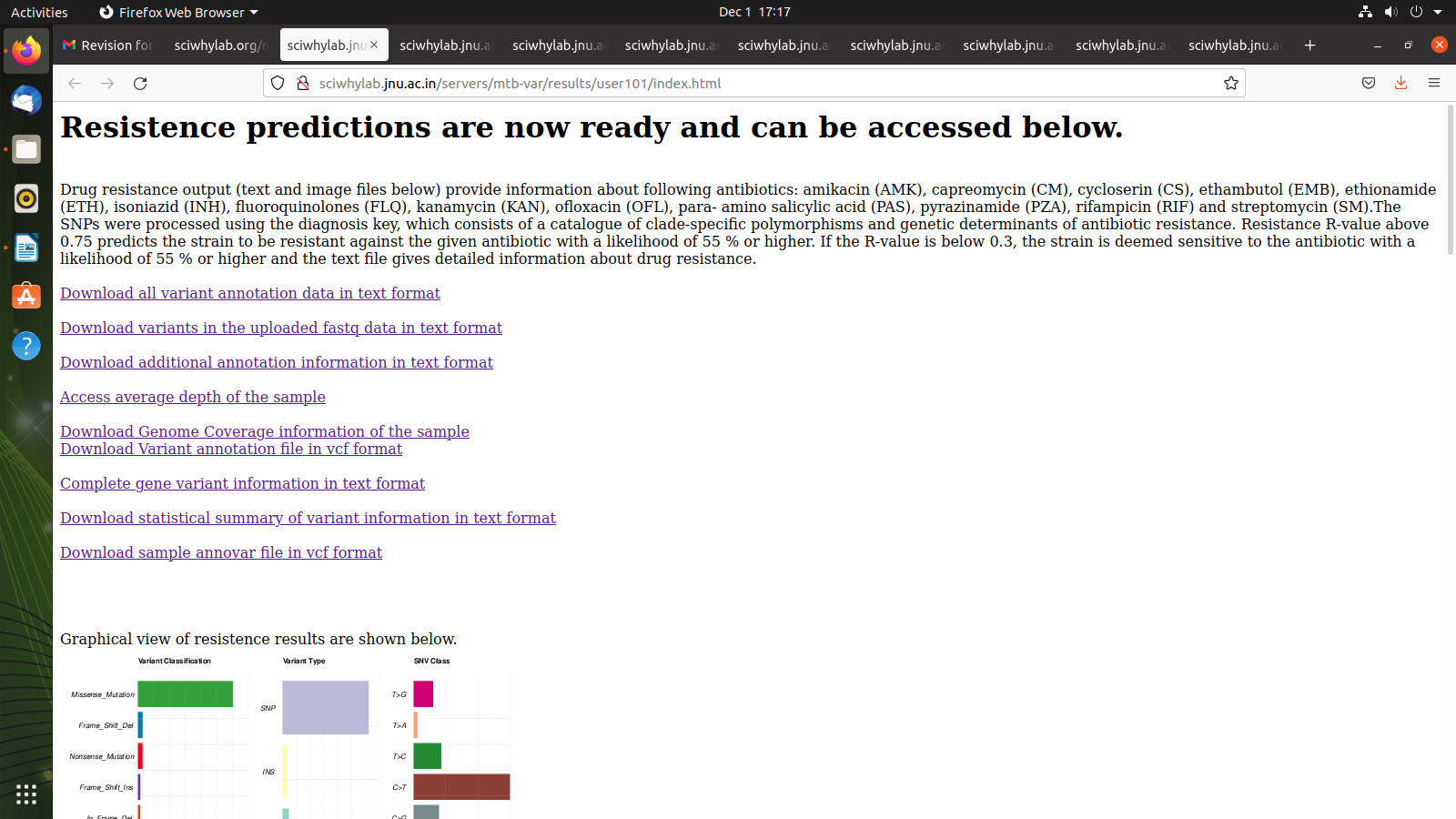
**Output pages of MycoVarP**

**Download all variant annotation:**


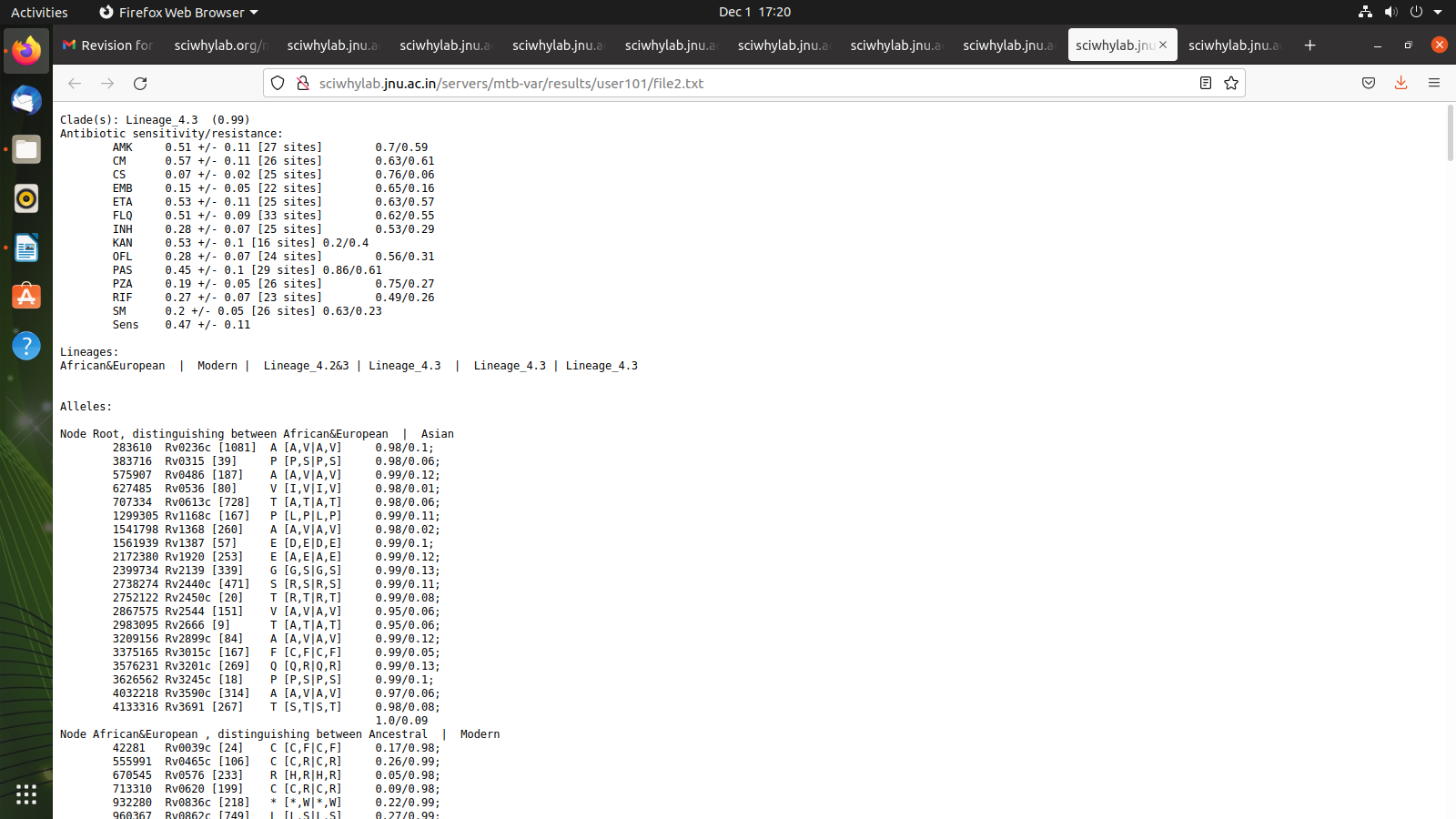


**Download additional annotation information:**


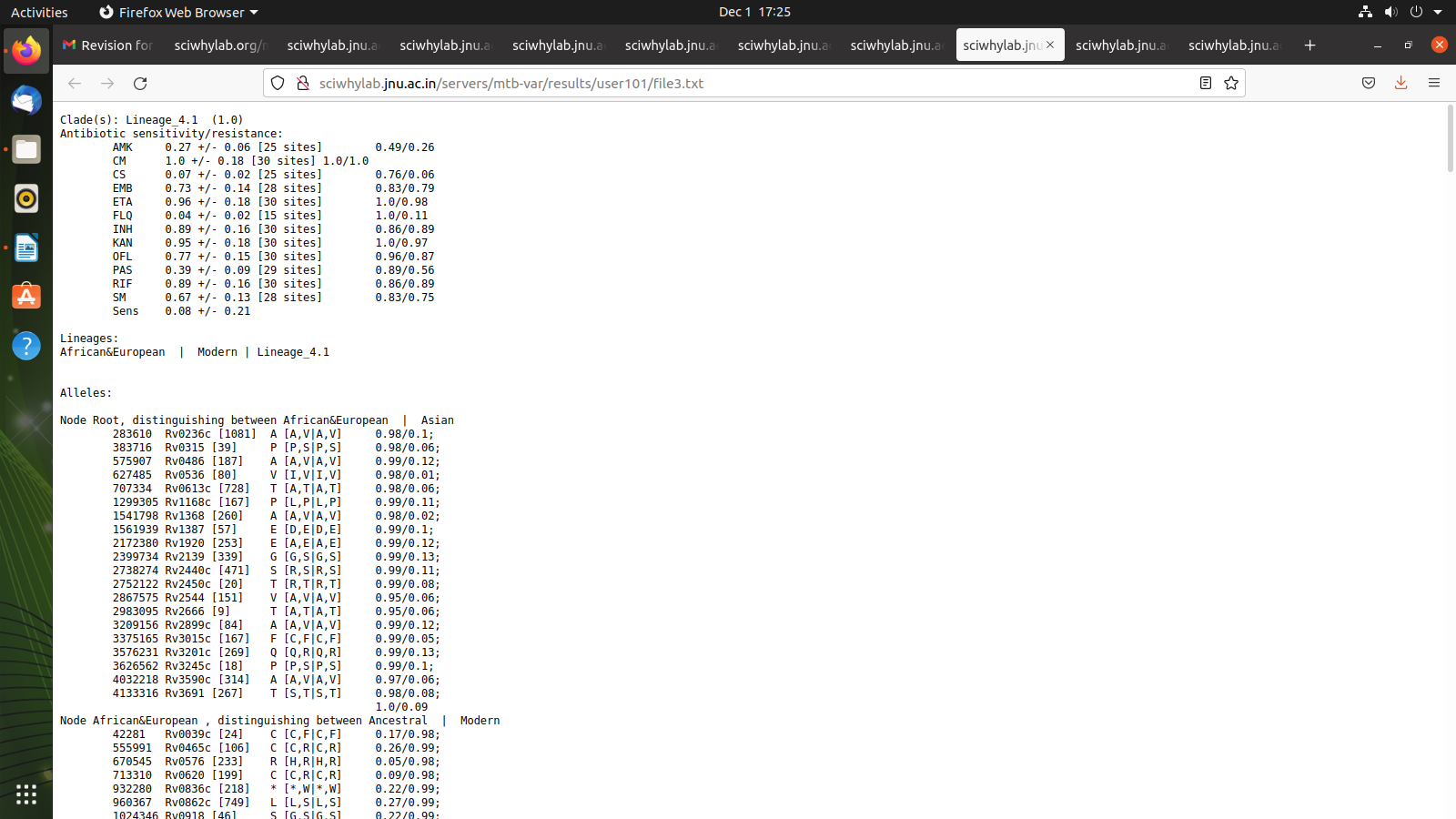


**Access average depth of sample:**


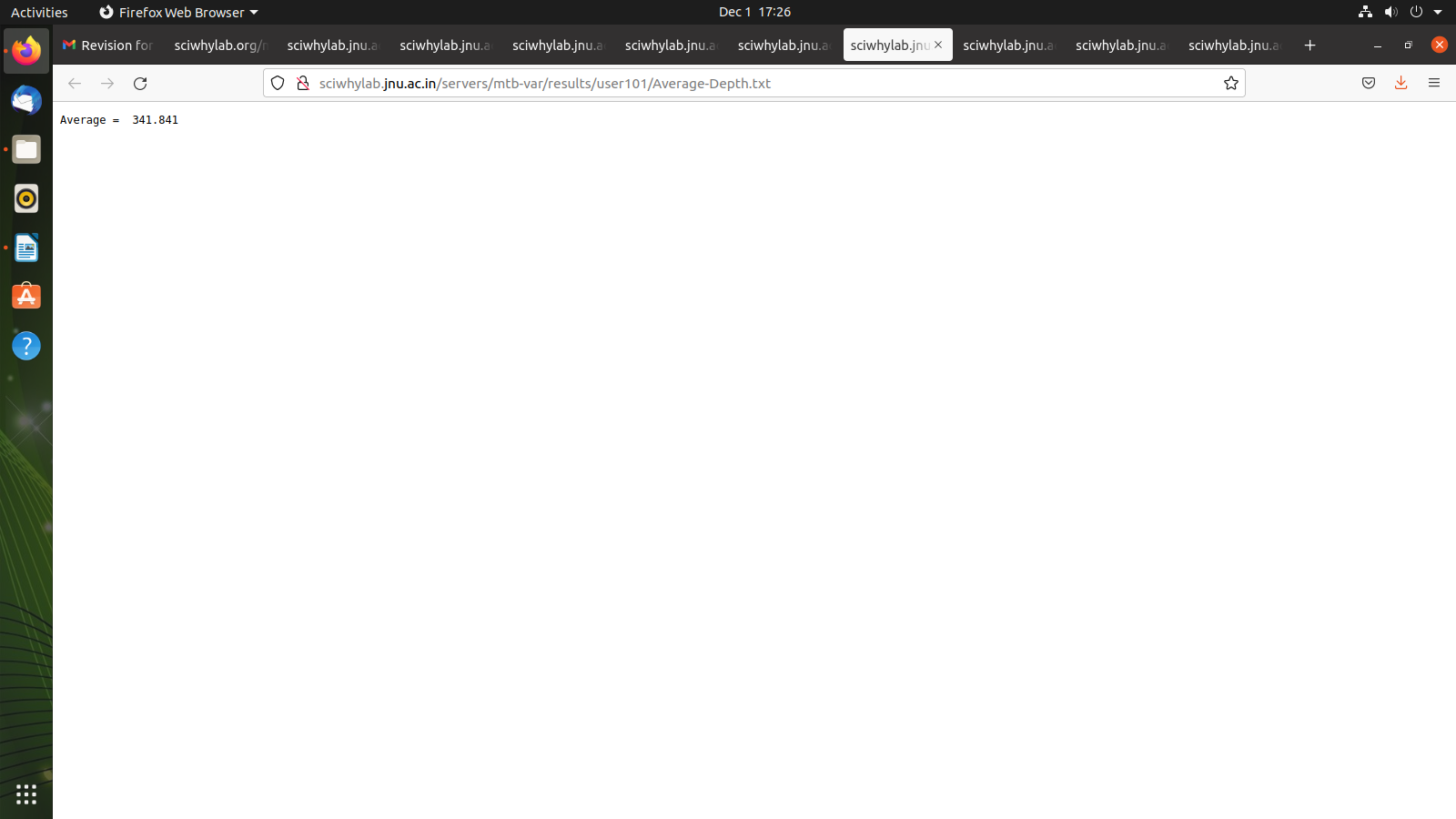


**Access Genomic coverage of sample:**


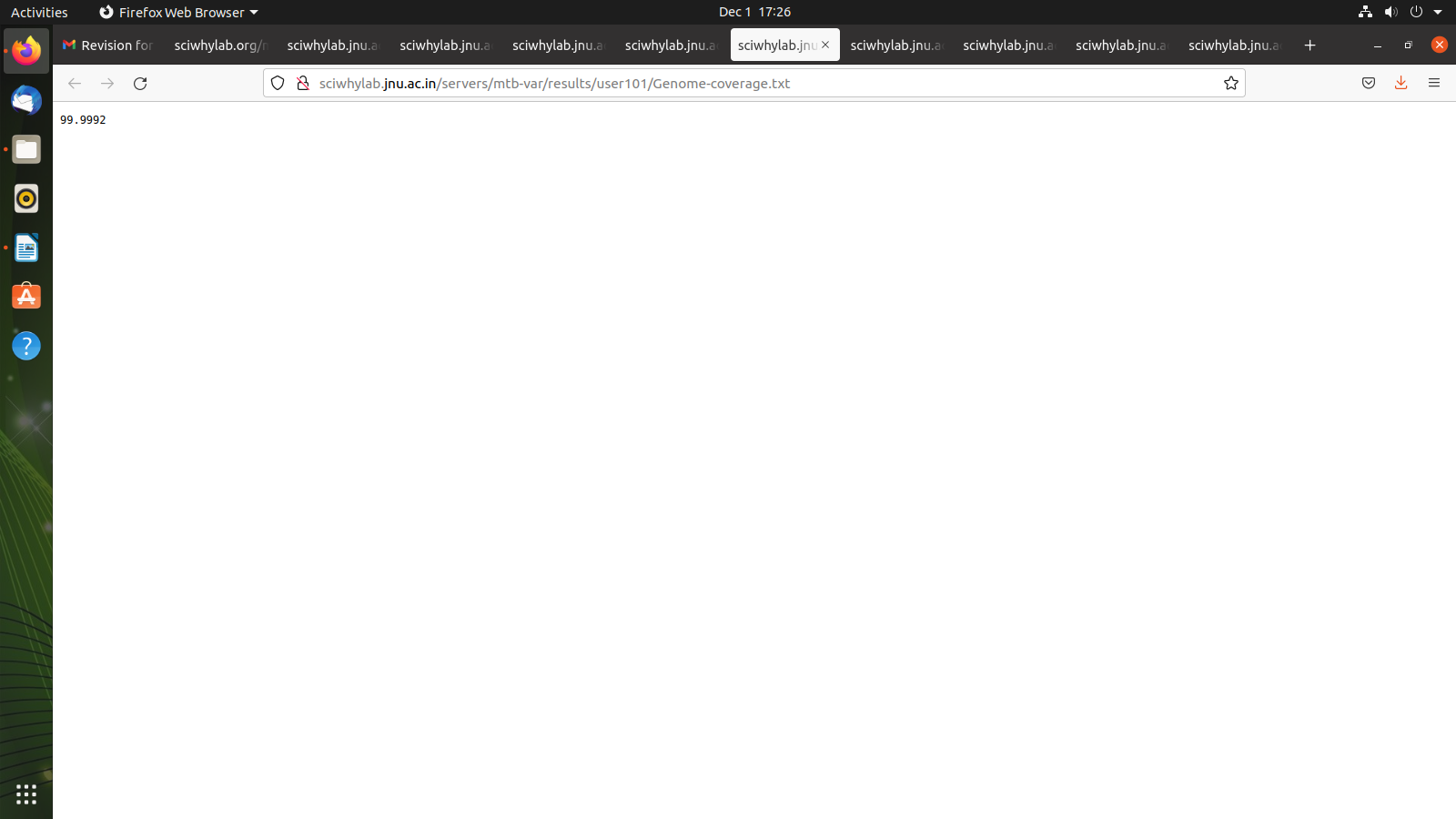


**Complete gene variant information**
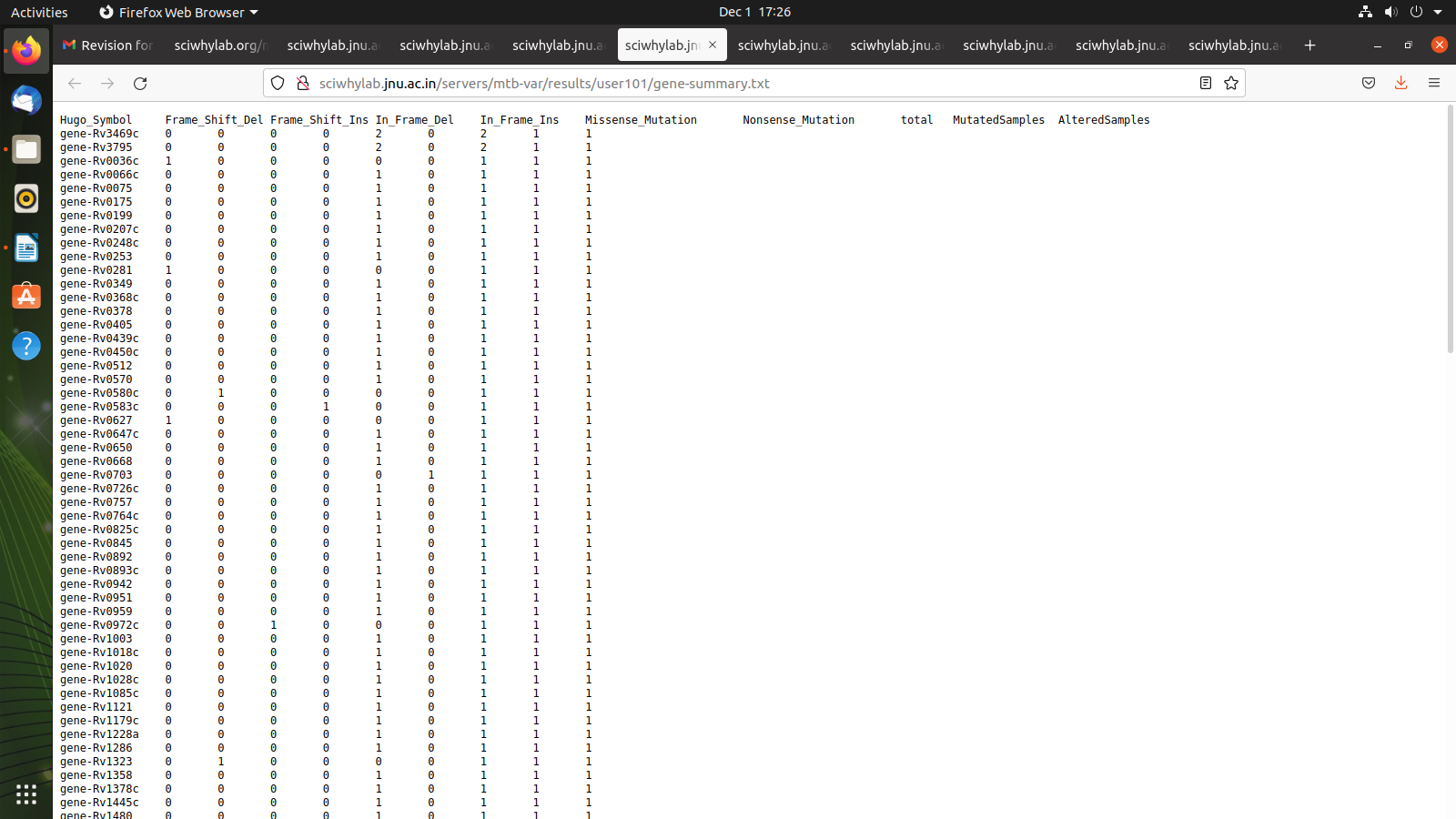
**:**

**Statistical summary of variant information:**


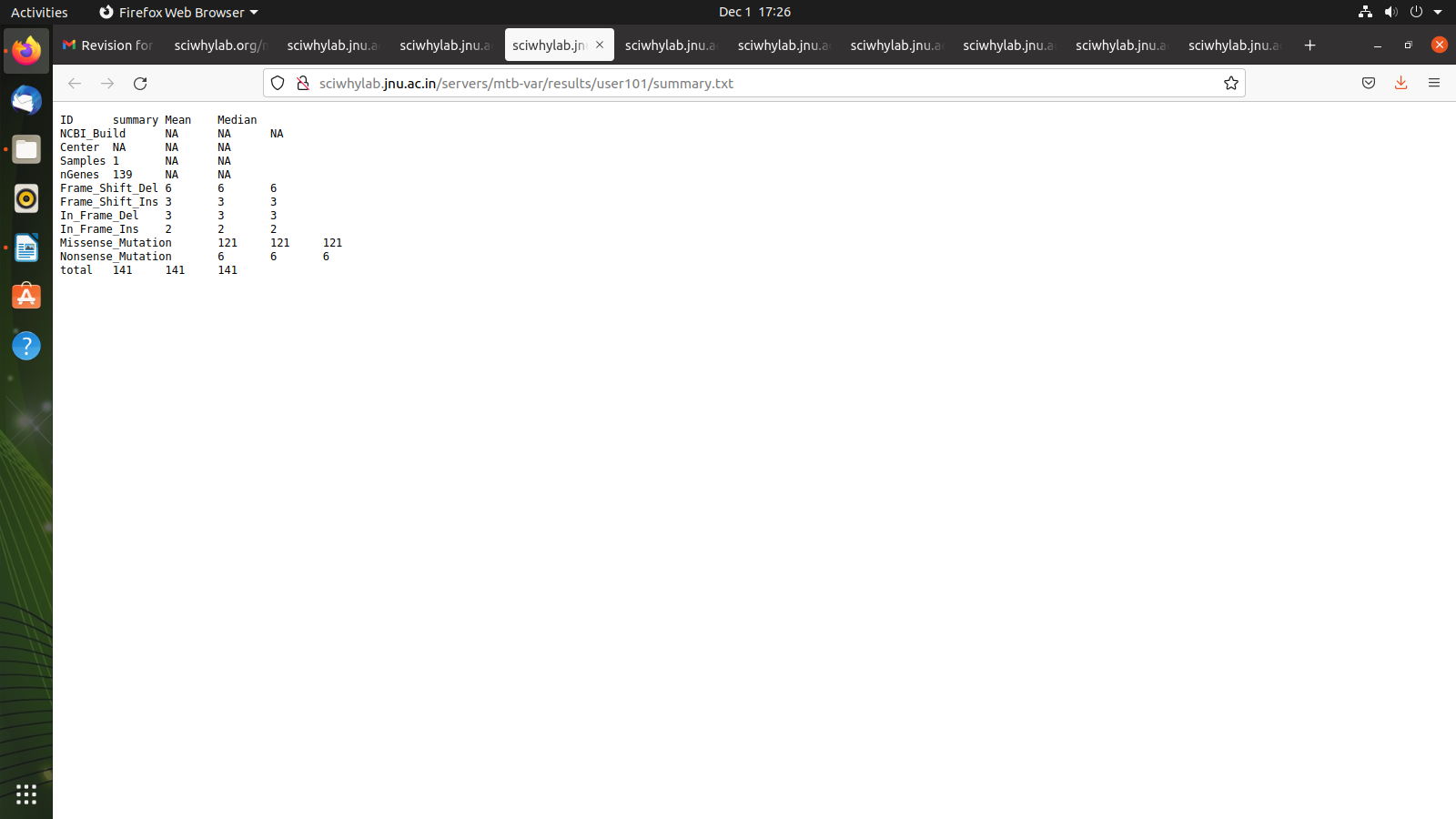


**Gra**
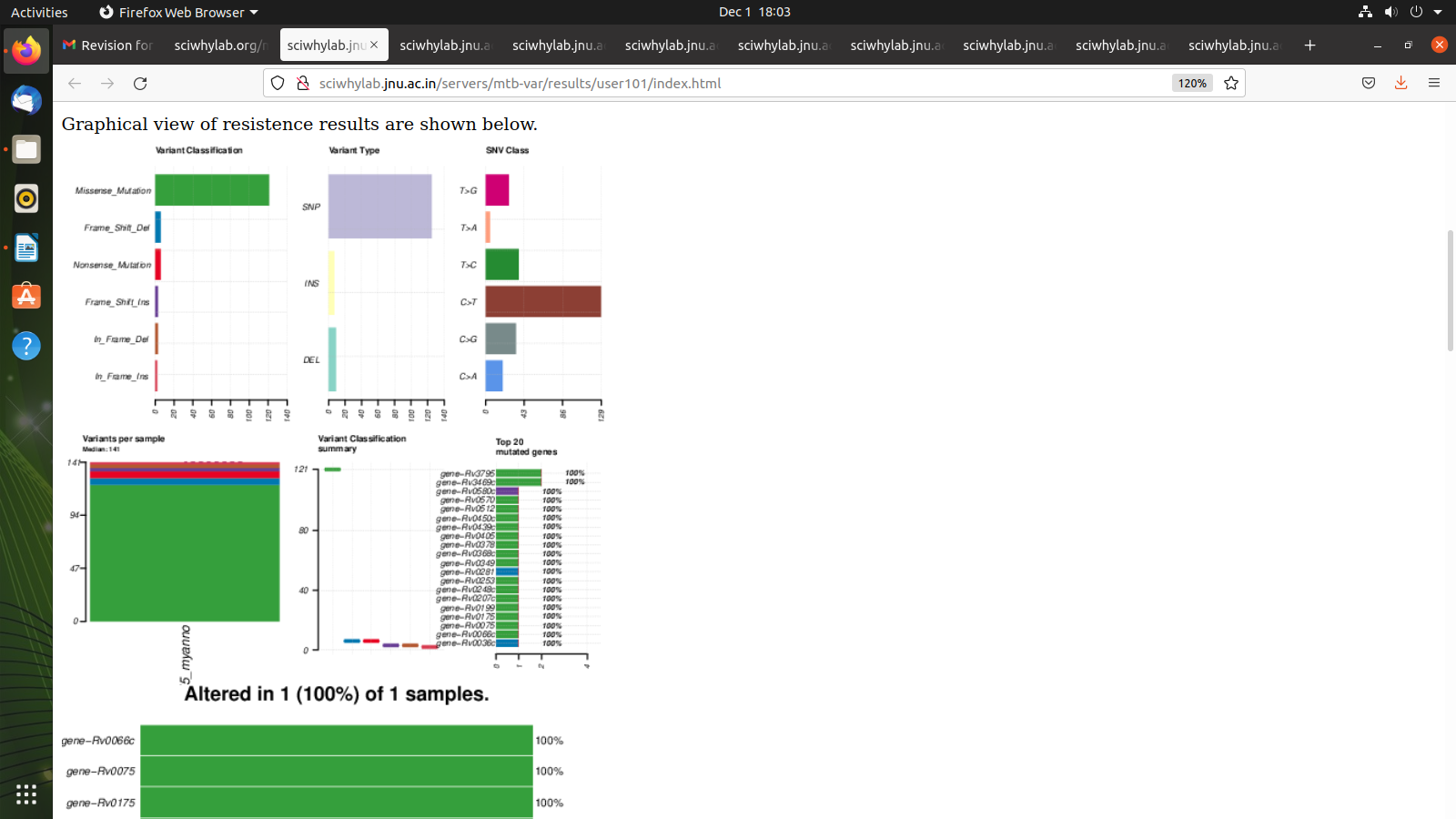
**phical view of resistence results:**


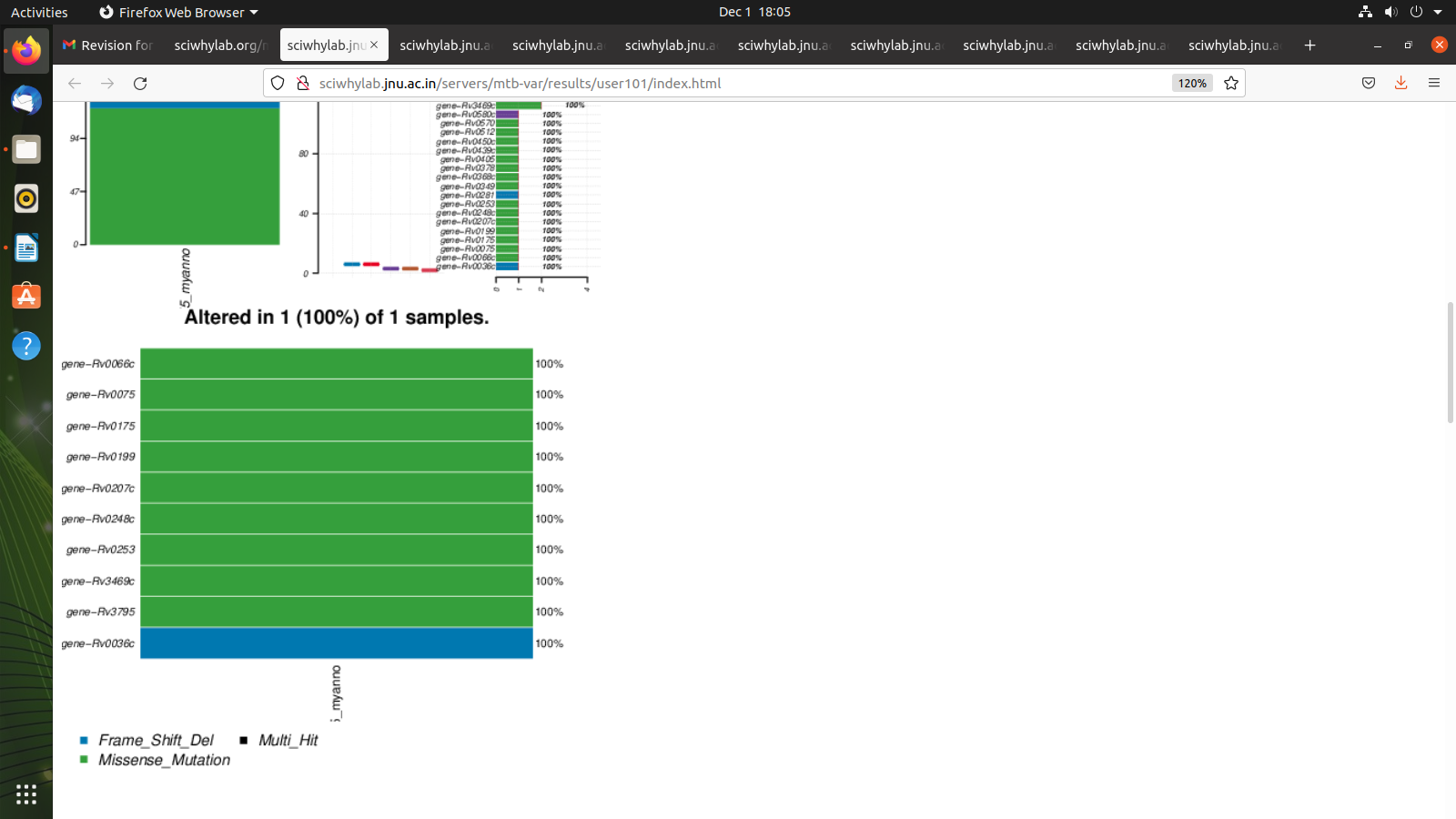


**V**
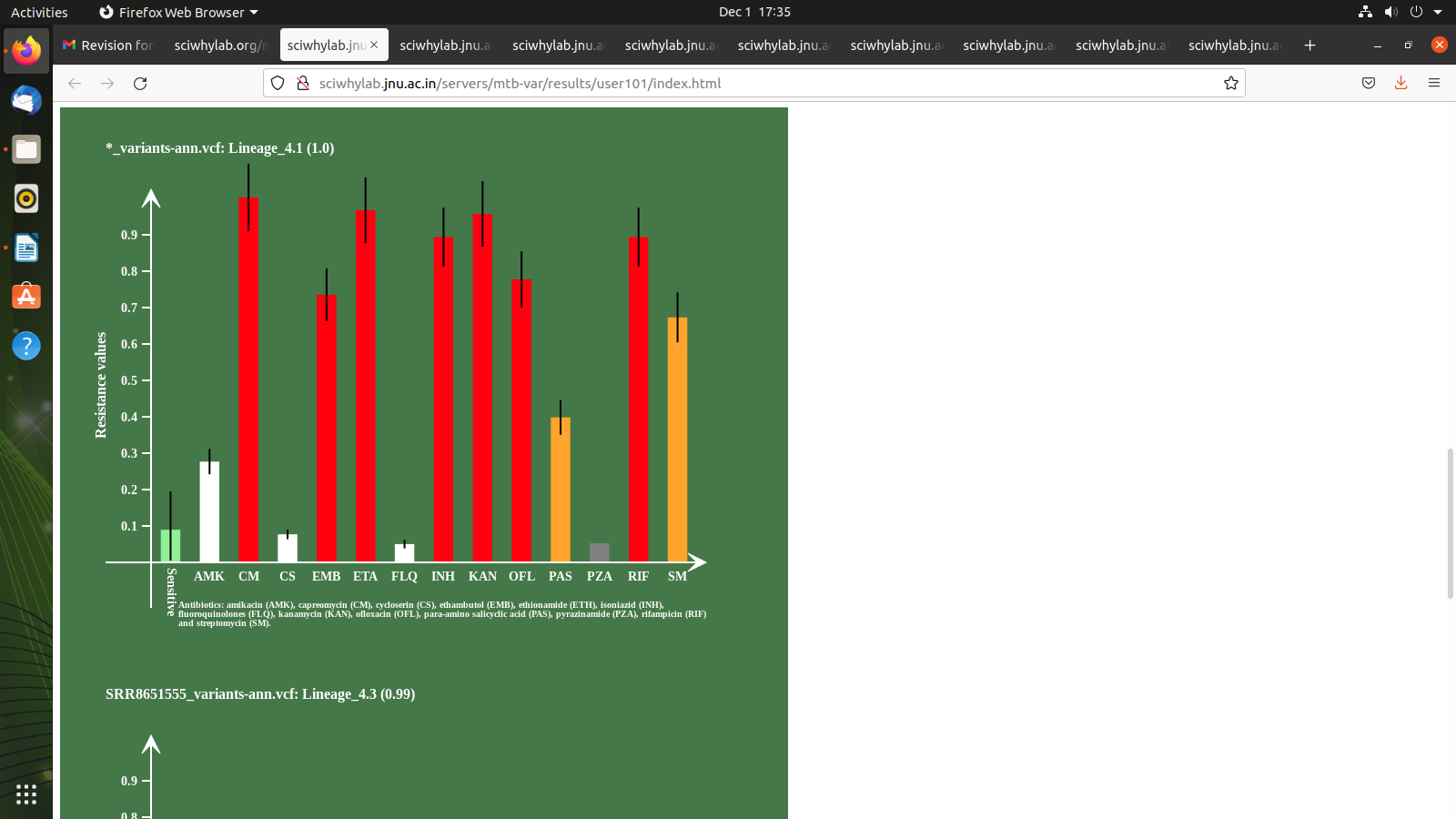
**ariants-ann.vcf Lineage_4.1(1.0):**

**Variants-ann.vcf Lineage_4.3 (0.99):**

**
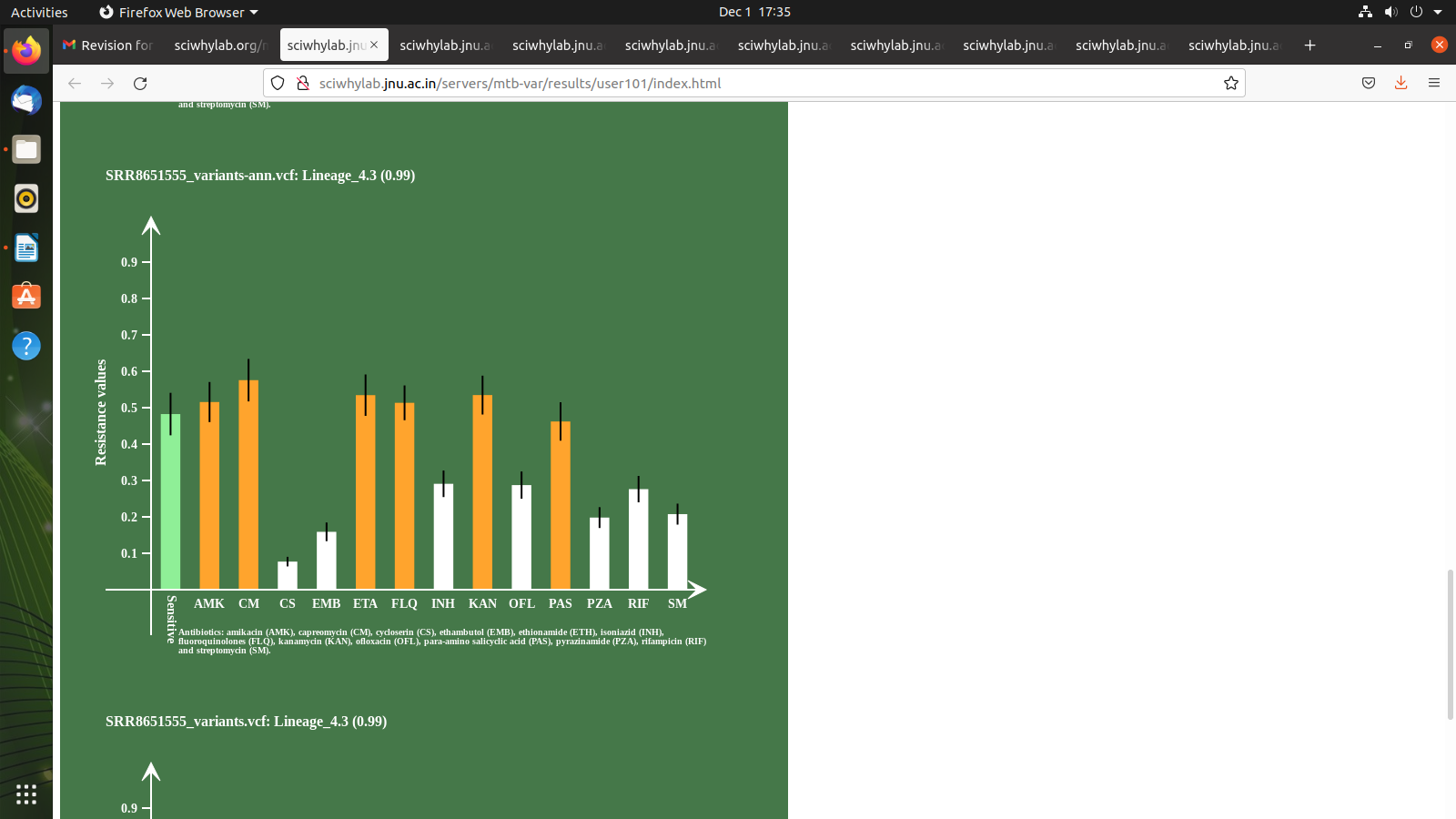
**
