# Supplementary figures and images for: MycoVarP: Mycobacterium Variant and Drug Resistance Prediction Pipeline for Whole-Genome Sequence Data Analysis
Source: Front Bioinform. 2022 Jun 3;1:805338. doi: 10.3389/fbinf.2021.805338 (PMC9580932; doi:10.3389/fbinf.2021.805338)

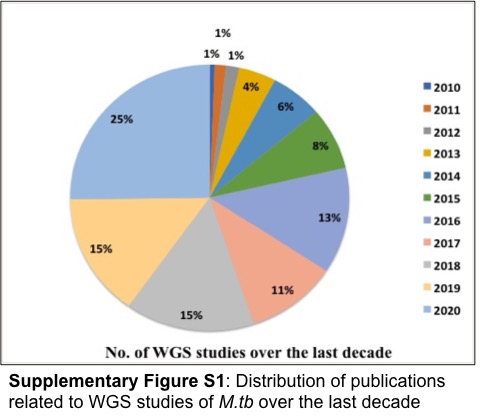

Supplement: Supplementary file 1 [file DataSheet1.zip › Supplementary Figure S1.jpg]
